# Diffusion Dynamics of Energy Saving Practices in Large Heterogeneous Online Networks

**DOI:** 10.1371/journal.pone.0164476

**Published:** 2016-10-13

**Authors:** Neda Mohammadi, Qi Wang, John E. Taylor

**Affiliations:** 1 Department of Civil and Environmental Engineering, Virginia Tech, Blacksburg, VA 24061, United States of America; 2 Department of Sociology, Harvard University, Cambridge, MA 02138, United States of America; 3 School of Civil and Environmental Engineering, Georgia Institute of Technology, Atlanta, GA 30332, United States of America; Tianjin University of Technology, CHINA

## Abstract

Online social networks are today’s fastest growing communications channel and a popular source of information for many, so understanding their contribution to building awareness and shaping public perceptions of climate change is of utmost importance. Today’s online social networks are composed of complex combinations of entities and communication channels and it is not clear which communicators are the most influential, what the patterns of communication flow are, or even whether the widely accepted two-step flow of communication model applies in this new arena. This study examines the diffusion of energy saving practices in a large online social network across organizations, opinion leaders, and the public by tracking 108,771 communications on energy saving practices among 1,084 communicators, then analyzing the flow of information and influence over a 28 day period. Our findings suggest that diffusion networks of messages advocating energy saving practices are predominantly led by the activities of dedicated organizations but their attempts do not result in substantial public awareness, as most of these communications are effectively trapped in organizational loops in which messages are simply shared between organizations. Despite their comparably significant influential values, opinion leaders played a weak role in diffusing energy saving practices to a wider audience. Thus, the two-step flow of communication model does not appear to describe the sharing of energy conservation practices in large online heterogeneous networks. These results shed new light on the underlying mechanisms driving the diffusion of important societal issues such as energy efficiency, particularly in the context of large online social media outlets.

## Introduction

There is a general consensus among researchers in the field that our planet is already experiencing the adverse impacts of climate change, with around 98% of climate scientists agreeing that these impacts are likely due to human activities [1]. This position is endorsed by nearly 200 of the world’s leading scientific organizations [2, 3] and the White House Climate Action Plan refers to humans as being “in the driver’s seat of the climate change system” [4]. However, public perceptions of the problem are less clear-cut [5, 6]. In a study by Leiserowitz et al. [6], 56% of the American public did not agree that global warming is primarily due to human activities. In the same study, 69% of participants mentioned that they needed more information in order to form a firm opinion about global warming; and only 6% said that “humans can reduce global warming and will do so successfully” [6]. Given that any serious attempt to significantly decrease the negative effects of human induced climate change will require vast and rapid societal responses [7], this substantial knowledge gap in public awareness is likely to stall efforts to move towards a more sustainable future [8].

Of particular concern are the increasing levels of greenhouse gas (GHG) emissions from human activities, which are the primary drivers of climate change [9]. In the United States, up to 84% of these emissions are reported to be energy-related [10] and world energy consumption is projected to increase to 56% by 2040 [11] as the scale of human activities continues to expand [12]. Surveys reveal that the American public underestimate the amount of energy they use by a factor of 2.8 [13], water by a factor of 2 [14], and potential household energy savings by a factor of 3 [15], largely as a result of their lack of awareness or access to the trustworthy information that is essential for decision making. This level of ignorance regarding even the most basic energy saving activities is directly linked to the lack of public support for climate policy [16, 17]. If we fail to deliver the relevant scientific and practical information in a readily comprehensible form, we cannot expect people to make informed private or public decisions in support of policy [18]. Likewise, without adequate public support and policy adoption, even the greatest technological advances or policy interventions will fail to address the targeted mitigations. Achieving substantial gains in energy efficiency depend on public decisions and policies [19]. Therefore, we need carefully targeted initiatives that increase public awareness and enable citizens to make more informed decisions leading to efficient actions such as the early adoption of new, more energy efficient technologies and using existing technologies more effectively. This has the potential to result in substantial environmental benefits [13], saving up to 123 million tons of carbon per year after only 10 years [20]. Improving the delivery of information and building awareness is a first step in engaging public support for these policies.

### Information Communication through Online Channels

The total number of internet users has now passed 3 billion globally, with around 3.8 billion Google searches, 200.9 billion emails, 7.8 billion YouTube videos, 1.3 Facebook active users, and nearly 700 million microblogs (“tweet”) posted on Twitter every day [21]. Online communication plays a major role in people’s choices and decisions and the internet has become the primary source of knowledge for many people, with online social networks offering alternative communication channels. Scientists are facing a dilemma, as until applied research is carried out to investigate how science is best communicated online, the dynamics of online communication systems are likely to have a far stronger influence on public perceptions of scientific topics such as climate change [22] than the avenues conventionally used by researchers to disseminate their findings [23]. Thus, those seeking to increase public awareness and perceptions regarding the need for energy saving measures need to adapt to become more focused on these new communication channels. Not only the information itself, but also who the information comes from, will play a significant role in these efforts because people often make choices and decisions based on the information they receive from their social contacts [24].

### Communication Theories, Channels and Complexities

Sharing information on energy-related issues as well as the influence of different channels of communication are among the most effective elements in models of decision making in behavioral interventions [25]. Communicating this information through online channels is thus a key factor in the effective diffusion of energy saving practices, and fundamental to efforts to attract public support for new energy policies. When using information as an instrument to promote energy conservation, it is essential to examine how it is conveyed [26] and through whom it is being spread, especially when widespread dissemination and effective public opinion change is desired. Although the existing literature has examined various channels of energy information delivery independently, it has largely overlooked the interoperability of these channels in today’s large online communication networks, which often combine a number of different channels of information delivery [27]. Thus, insights about the complexity of this process are crucial if we are to develop a better understanding of the diffusion of energy saving practices.

In the 1930s, the most widely accepted communication theory, the so-called Hypodermic Needle theory [28], contended that the media has a direct effect in informing and changing public opinion. A few years later Lazarsfeld introduced the Two-step Flow of Communication theory [29], which suggests that individuals change their behavior or form an opinion once influenced by “opinion leaders” who are, in turn, influenced by mass media appeals. The flow of ideas is thus considered to pass from mass media to opinion leaders to individuals, with the greatest influence being the indirect and personal influence of opinion leaders on the wider population. While this model has been both disputed and supported in the years since, the essential hypothesis has remained valid and a comprehensive framework based on this theory has recently been presented that illustrates how a combination of social network and human social motive elements is required to enable the diffusion of scientific discoveries to the public at large [30]. Studies of online communications suggest that nearly 50% of the information spread by media is diffused indirectly through opinion leaders to the individual users, who are themselves classified as individuals, yet are further connected, and exposed to the media than others [27]. It has also been suggested that some online social networks have properties that are close to those of a news media platform [31].

Embedded in these communication theories, successful channels of energy information communication are traditionally identified as either “one-way” mass media appeals or “two-way” interpersonal communications [32] of the type often referred to as Word-of-Mouth (WOM). One-way communications often follow a standard broadcasting mechanism and have been employed in home energy audits, energy information transmissions in workshops and mass media campaigns [33], as well as for raising public awareness through commercials within larger networks [34]. Two-way, or interpersonal delivery of energy information, is known to be an effective way to raise awareness of new products and ideas, and persuasive in changing opinions and behavior [35, 36] in social diffusion such as marketing [37], elections [38], and innovation adoption [39], primarily due to social reinforcements from others in an individual’s social network. WOM, by definition, is the act of individual to individual information exchange and it is important to distinguish this mechanism from the marketing concept Word-of-mouth Marketing (or Word-of-mouth Advertising), which is an intentionally encouraged marketing plan. WOM is instead an unbiased communication between individual receivers and communicators who are known to them and whom the receiver regards as making a neutral assessment of a product, brand or service [40].

Communications in online social networks cannot be studied by simply focusing on a single channel, as they are designed to facilitate direct and reciprocal communication links between the mass media, opinion leaders, and the public. This means that studies of social influence that focus on local and endogenous processes such as WOM are open to charges of neglecting important exogenous effects such as marketing or mass advertising, and efforts to separate these two confounding components in today’s complex and heterogeneous online social networks can be misleading [41]. While recent studies have carefully focused on the importance of social influence in examining the spread of ideas, practices, and behavior that target positive public impacts [30], they have generally underestimated the inextricably interwoven dynamics of large (in particular online) social networks, which are by nature heterogeneous, consisting of diverse entities who are directly connected and are able to communicate with each other. The time-dependent dynamic behavior [42, 43] that such circulating communications exhibit across different entities adds to the complexity of these networks. While online social networks offer unparalleled opportunities for large scale social diffusion, the underlying processes responsible for propagating such communications and their subsequent influence, if any, require empirical studies and their results are poorly understood. We lack empirical evidence that unambiguously demonstrates how online social networks articulate the successful diffusion of energy saving practices.

This study focuses on studying the diffusion of energy saving practices through an online social networking platform, analyzing the tweet/retweeting behavior of communicators and tracking communications on energy saving practices. The participating entities are classified as organizations, opinion leaders or public individuals, and the flow of communication across these entities is tracked by mapping their social networks onto their communication networks and building a who-told-whom network of energy saving practice communication. In order to better understand the diffusion dynamics, we posed the following questions. First, to investigate the underlying dynamics involved we asked: *can the diffusion of energy saving practices in such networks be formalized as epidemics or innovation diffusion in terms of transmissibility and are the underlying dynamics of these networks endogenous or exogenous?* Next, we examined the communication process itself, asking: *what are the dominant communication processes involved in energy saving practice diffusion in such networks (i.e. mass, personal, or interpersonal)?* Finally, to explore the effect of influence on propagation, we asked: *who are the most influential entities in the network, and who has the greatest influence on the intended target audience (i.e. the public)?*

We examined the two-step flow of communication in the context of energy information flow and influence, where entities share energy saving practices learned from in network with others in the network. For each user, we calculated the proportion of their social contacts to whom they diffused the energy saving practices further and measured its value both in breadth and depth within the communication network to serve as a measure that approximates their level of influence in propagating energy saving practices. The level of influence for each individual in terms of the breadth of the communication network is referred to as a *propagation-score*, and in terms of the depth of the communication network a *hop-score*.

Heterogeneous social networks, in which various entities with a variety of social statuses, are able to directly communicate with one another [44], have undermined the efficacy of mass vs. interpersonal communication dichotomy [27], and likely require a heterogeneous view of the communication pattern rather than a traditionally segregated view [45]. One example of such heterogeneous networks are online task-oriented social networks (TOSNs), in which circulating communications related to different activities occur between different types of entities. Such communications alternate over time and create temporal motifs that are related to distinct dependencies between entities [46]. Thus, a single communication channel such as the two-step flow of communication, which has been successful in real-world cases [29], may not necessarily be the dominant flow of communication in such networks. Likewise, as revealed by the science of networks [30], or expected from real-world, or online [27] cases, opinion leaders may not necessarily be the most influential entities of the network, particularly in specific contexts like energy saving practices. It follows, then, that the public may be being mostly influenced by these individuals. Therefore, we posed the following hypotheses which guided our examination of the patterns of diffusion in our energy saving practice diffusion network:

#### H1

In diffusion networks of energy saving practices, the communication pattern is heterogeneous across organizations, opinion leaders, and individuals; and the two-step flow of communication theory is not necessarily the dominant flow of communication.

#### H2a

Mass personal communicators (Opinion Leaders) are not predominantly the most influential entities in diffusion networks of energy saving practices.

#### H2b

Interpersonal communicators (Individuals) are not predominantly influenced by Mass personal communicators (Opinion Leaders) in diffusion networks of energy saving practices.

## Methods

### Data

We conducted our study by collecting data from Twitter, a social networking platform [47]. In the initial phase, we collected energy information sharing activities along with social ties information in three steps based on Brown and Reingen’s approach [48], through the public Twitter Stream API [49]. First, we streamed time-stamped tweets that are related to energy conservation by filtering the tweets in real time for a list of phrases such as “save energy”, as illustrated by Wang and Taylor [50]. The data collection lasted for 28 days between September and October 2012 and a total of 108,771 tweets were collected. Tweets collected in this way contain content information such as Tweet IDs, Twitter end user profile information, etc. Second, using multiple keywords, we filtered the raw data to ensure they were strictly related to energy saving practices, this time using a natural language processing module (NLP) [50, 51]. A total of 1236 Tweets, and 1,084 Twitter end users, yielding 256 original energy tweets plus 980 replicated tweets (retweets), were observed within the communication network. Lastly, we retrieved the social relationship data for all 1,084 Twitter end users at the end of the 28 days [50]. In the second phase, we retrieved basic information for all the 1,084 Twitter end users, including their profiles, location, and their numbers of followers, followings, and posts. As stated earlier in this study, the social contacts from whom people receive their information are critical in their awareness and decision makings [24]. To explore this argument in terms of energy saving practices, we classified the retrieved end users (entities) as organizations, opinion leaders, or individuals. The content of this dataset are directly associated with end users account information and subject to confidentiality restrictions. The datasets, therefore, remains in compliance with Twitter’s non-disclosure agreement.

### Who-told-whom Network of Communication

In order to determine patterns of diffusion for energy information among different types of entities, we created a ‘who-told-whom’ network of communications between the three entities of organizations, opinion leaders, and individuals using the ‘following’ relationship network data for the same entities.

**Fig 1:**
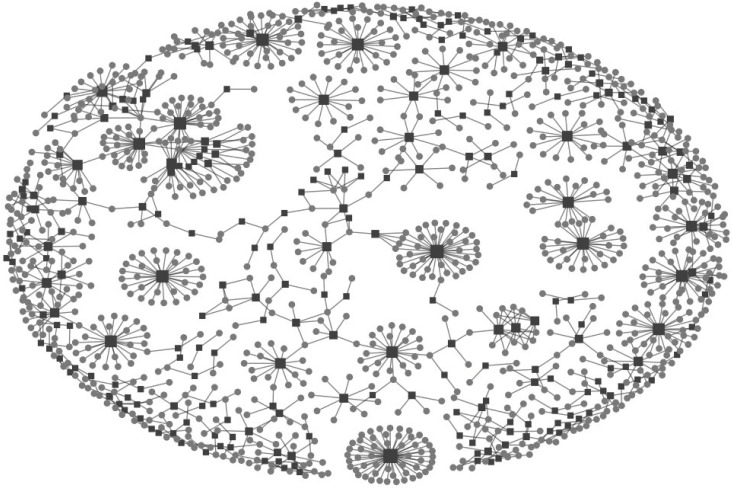
WOM Affiliation Network. Circles represent each entity (*E*), and squares represent the energy tweets (*e*_*k*_). The size of each square node is proportional to the number of connections to that node.

Fig 1 depicts the communication network that emerged from this energy information sharing.

**Fig 2:**
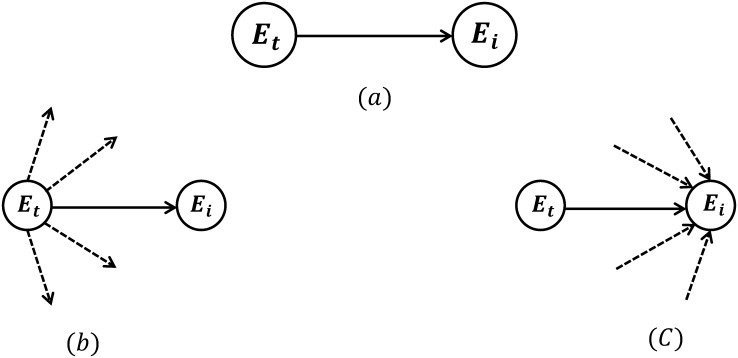
Following relationship. In-degree: (a): *E*_*t*_ follows *E*_*i*_. (b): *E*_*t*_: active follower when retweeted from *E*_*i*_. (c): *E*_*i*_ influential followee whose tweet got retweeted by *E*_*t*_.

We consider this network an affiliation network within which the entities are affiliated with the same energy tweet if they have participated in sharing that information in the network. The unique structure of Twitter’s articulated connections only allows for defined ties and dissemination paths which correspond to its network structure. The topology of the Twitter network dictates the diffusion phenomenon through the entities’ social ties. Fundamentally, social ties in Twitter can form without requiring approval from the other party and are not necessarily reciprocal. We examined three different combinations of social ties [52] in Twitter: contact-perceived, when a contact follows an entity; subject-perceived, when a contact is being followed by an entity; and mutually-perceived, when the entity and contact both follow each other in a reciprocal relationship. Here, we only considered one way following relationships (i.e., in-degree)–a directed social relation in which an entity is followed by a contact (a contact follows an entity)–as shown in Fig 2(a). This allowed us to measure the influence of an entity over its contacts.

We explored the extent to which each entity type participated in the diffusion of energy saving practices, the extent of their influence, and the distribution of the diffusion flow among the entities. To understand the underlying mechanism of diffusion around energy topics, we implemented several steps to analyze the data. We observed that the diffusion of energy information in online social networks is actually a multi-centric epidemic with multiple sources, rather than just one point source [53]. To identify these sources and their associated epidemic properties, we divided the social network into different groups corresponding to the 256 different energy tweets in the sample, placing the communicators involved in the diffusion of each energy tweet in the same group. In each group, one communicator acts as the initiator by posting a piece of energy-related information i.e. an ‘energy tweet’, which is then picked up by entities who follow that communicator who repeat-post the same information, i.e. an ‘energy retweet’. Further, we classified all entities as either ‘Organizations’, ‘Opinion Leaders’, or ‘Individuals’ and evaluated a number of their attributes such as occupation, number and nature of followers, number and nature of followees, and number and nature of tweeting activities. Through manual screening, a thorough qualitative and quantitative evaluation of the data around these attributes for each non-organization entity [29, 54] was performed that enabled us to classify them as follows:

*Organizations*: all the businesses, companies, communities, and organizations whether directly related to energy efficiency/conservation or indirectly promoting energy savings; those who have biased goals or are dedicated to sharing energy information are categorized in this group.

*Opinion Leaders*: these individuals have relatively high social status and a large number of social connections [55]. The number of followers has often been a prominent metric in determining one’s level of influence, which is considered to consist of the ability to reach a broad audience [56, 57]. However, in order to decide whether an individual has established credibility within his/her network and can be regarded as an opinion leader, we also incorporated suggestions from Nisbet and Kotcher’s three dimensions for climate change opinion leaders [58], which follows the two-step flow of communication model. We included personal accounts affiliated with an organization in this group.

*Individuals*: Once we had identified the opinion leaders among the non-organization communicators, we classified the remainder of the communicators in this group.

For this classification, we pursued a data-driven approach. First, we precisely separated the ‘organizations’ from the rest of the entities based on the clear distinction between an organizational vs. personal (non-organizational) account. Next, we sorted the accounts into either opinion leaders or individuals. Several metrics have been suggested to identify various online communicators [59, 60] and evaluate popularity and influence, such as a user’s follower count [61, 62], followers/followees ratio [62, 63], tweets per follower ratio [64], retweet count [62, 65], Page Rank [61, 66]and so on. We adopted ‘popularity’ as our metric, regardless of that entity’s general level of influence. Ideally, we aimed to evaluate whether an entity advocated any context-specific influence (i.e., energy saving practices) in particular. Several structural properties of online networks are used to represent an established reputation. Xuan et al. adopted node degree (incoming and outgoing), Page Rank, and Hits to rank developer candidates as well as identify emergent developers in OSS projects by examining their social links [66]. Kwak et al. ranked the entire Twittersphere (41.7 million user profiles) using both number of followers and Page Rank to determine the popularity of each account and found the two rankings to be similar [61]. To this end, using the popular metric of end user’s follower count, we extracted from personal (non-organizational) accounts, those whose number of followers exceeded 2000 and identified them as opinion leaders. In choosing the user’s follower count of 2000 as a threshold, we refer to Twitter’s 2000+ following limit rule [67], and added the requirement of: maintaining a minimum followers-to-followees ratio of approximately 1 for a genuine account with high reputation [62] (i.e., at least around 90% ratio is required before being able to follow further users) to our analysis. Our observations align with the findings of previous studies, who reported that the largest cascades of information are generated by users with a larger number of followers [68].

To identify the flow pattern for each energy tweet, we aggregated all the entities who posted a similar tweet, and traced the path through which that tweet traveled corresponding to the various social ties. We treated every tweet as one communication activity and every retweet as a sign of effective energy information diffusion (i.e. influence spread). Retweeting refers to the behavior of re-posting tweets from another entity in the network [69], which implies that one specific tweet has caught the attention of others [56] and led to further action. In other words, an entity is acknowledging the importance and value [68] of one piece of energy information, and has taken the action of spreading that information further through his or her own social contacts. Thus, we consider retweeting to be a measure of influence. We are confident that an entity is influenced by the information received if the receiver of that information decides to propagate it further.

**Fig 3:**
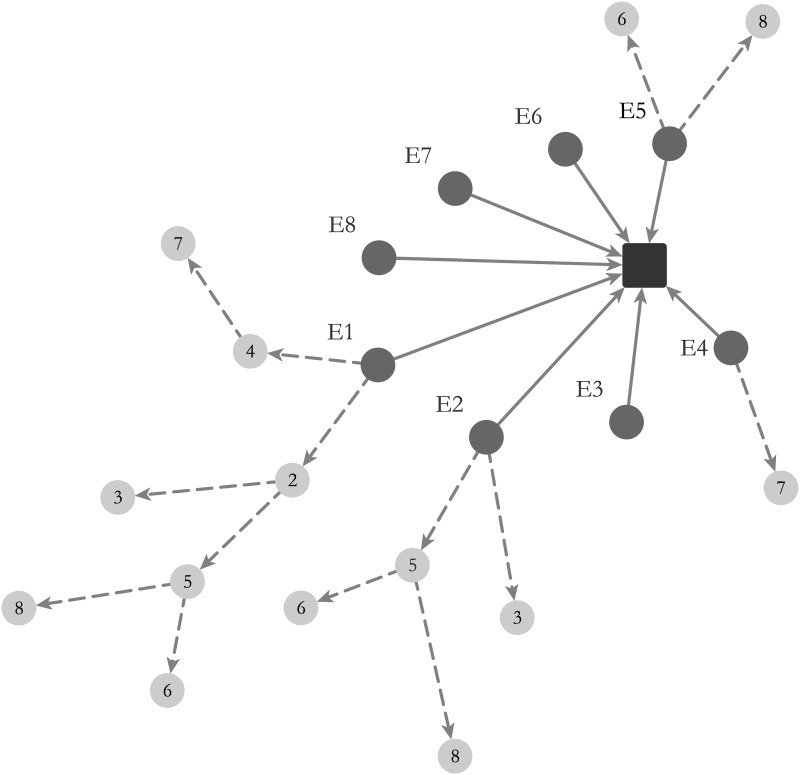
Who-told-whom network for energy communications.

We quantified the propagation of energy tweets corresponding to the social ties of the entities embedded in the diffusion network and then analyzed the contact-perceived relationships through which a contact (follower) identifies an entity (followee) to follow. Every follower will observe the energy tweets initiated or retweeted by one of his or her followees. We screened the time stamped retweets for each energy tweet, and superimposed these onto the social ties network to produce a ‘who-told-whom’ network of communication for energy information, as illustrated in Fig 3.

Assumptions made in this analysis are: (1) retweeting is only done by an ‘active follower’ (Fig 2(b)). This excludes retweets from: public streams, trending topics and explore tabs, and supports our argument by excluding the un-connected retweeters from the network; (2) only retweeting is utilized as a sign of propagation (no conversation or @username referral is included), and (3) according to Twitter’s functionality, each entity will only see the earliest version of a post (tweet/retweet) among its followees (i.e. whoever retweeted first). Corresponding to the time-stamped order of tweets, there will thus only be one ‘influential followee’ (Fig 2(c)) for each entity, which implies no duplication in the following relationships per retweeting action. The who-told-whom network is denoted by *G* = {*E*, *e*_*t*_, *E*_*e*_*t*__, *E*_*E*_}, where *E*, and *e*_*t*_ are two type of node sets: *E* = {*E*_1_, *E*_1_, …, *E*_*N*_} are the entities (i.e., organizations, opinion leaders, and individuals) and *e*_*t*_ = {*e*_*t*_1__, *e*_*t*_2__, …, *e*_*t*_*K*__} are the energy tweets. *E*_*e*_*t*__: *E* → *e*_*t*_, and *E*_*E*_: *E* ⤏ *E* represent the communication links (i.e., tweets or retweets) between the nodes. *E*_*e*_*t*__ is generated every time an entity tweets *e*_*t*_; *E*_*E*_ is generated as a result of superimposing the social relationships network of entities over the network of *E*_*e*_*t*__: *E* → *e*_*t*_ links. In this process, the dark nodes will either remain an originator of energy tweet *e*_*t*_, if it does not hold any social link with other entities; or will turn to a grey node (i.e., active follower) if the act has been retweeting the energy tweet *e*_*t*_ based on a *E* ⤏ *E* following relationship. In this case, the initial dark node becomes and influential followee. These links are generated in the sequence of time that they have been retweeted. Fig 3 shows the superimposed state of this network for one energy tweet. The process is then repeated for all energy tweets and duplicate links are eliminated. To measure the degree of propagation, we introduced the notion of propagation-score. We identified retweeting frequency as a metric, and gave the entities higher propagation-scores if their action had reached further, defining propagation-score in terms of the total number of retweets which that entity has caused, i.e. the number of individuals whom the entity’s initial tweet has reached. We quantified this effect as follows:

for each energy tweet *et_k_*:

$$P_{i}=\sum_{j=1}^{Fi}f_{i,j}$$ (1)

$$f_{i,j}=\left\{\begin{array}{cc} 1 & \;\text{if}\;E_{j}\;\text{is}\;\text{an}\;\text{active}\;\text{follower}\;\text{of}\;E_{i}\\ 0 & \;\text{otherwise} \end{array}\right.$$

*P_i_ = Propagation-score for u_i_*.

F_i_ = Total followers of u_i_.

et_k_ = Energy tweet k.

The propagation-score identifies the most influential entities in the network. To identify the depth of their influence in the network, we measured the number of hops each communication activity traveled; where a tweet is propagated over three degrees of influence [19], this is taken to indicate a successful propagation of influence in our network and is measured by the hop-score for each entity in the network. Each user E with a solid tie is either an ‘initiator’ of that tweet (*E*_1_) or a retweeter. Dotted ties indicate the propagation network for each user based on their following relations. Lighter nodes represent the affiliated entities (active followers) of that energy tweet who are in their following relation network. Each user is assigned a propagation-score according to the number of his or her followers who are influenced by a retweeting action and a hop-score based on the number of hops traveled by their energy tweet. S1 and S2 Figs represent the positive skewed distributions for P-scores and H-scores among various entities with their statistics indicated in S1 and S2 Tables.

In our analysis, implicit propagation is not considered, and the network under study is static. Also, our social relation data may contain deviations due to the unavoidable data processing time-lag involved in collecting energy tweets and social relations sequentially due to Twitter’s data streaming limitation.

## Results

A total of 256 original energy-related tweets plus 980 replicated tweets (retweets) were generated by 1,084 Twitter users during the 28 day observation period. Interestingly, there was a significantly low density for the communication network of energy saving practices (x = 0.00136), and its corresponding social network [50]. This population was found to consist of the following communication demographics: 85% organizations, 4% opinion leaders, 7% individuals, and 4% inexpressive data including false data and blocked user profiles. The great majority of these communications were initiated by organizations. In terms of the share of tweets sent by different entities, 89% of the energy-related tweets were initiated by organizations, 3% were from opinion leaders, 7% were from individuals, and 1% were from inexpressive entities. In the case of the most active communicators within each category, 72% of the time organizations were retweeting, 8% of the time it was opinion leaders, 18% were individuals, and 2% inexpressive users.

**Fig 4:**
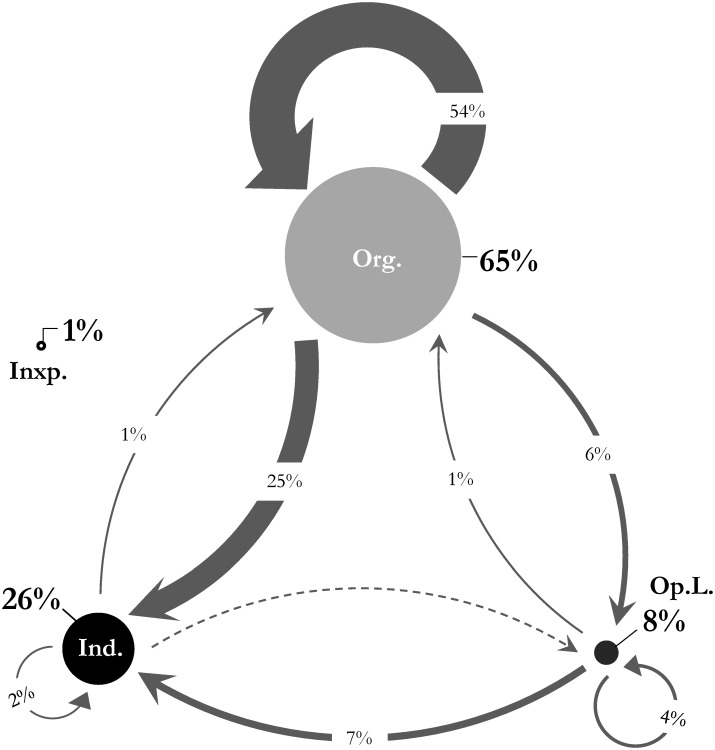
Diffusion Flow. Diffusion flow between three entities of: (1) Organizations (Org.), (2) Opinion Leaders (Op.L.), and (3) Individuals (Ind.). The direction of arrows represent the retweeting behavior and the thickness is indicative of the magnitude of flow.

Fig 4 tracks the diffusion flow pattern of energy saving practice communications between various entity types. The size of the sphere for each entity type represents the share of all tweets and retweets in our data sample. Organizations contributed to sharing energy information 65% of the time, opinion leaders 8%, and individuals 26%; around 1% inexpressive communications were also recorded. Directed links between entities represent retweeting behavior, meaning that a tweet has been retweeted from the initiator by an active followee (Fig 2(a): *E*_*i*_). Considering the possible trends of information flow and influence between our three pre-defined sets of entities, we observed eight out of nine possible linkages, with the missing energy information sharing link being from individuals to opinion leaders. Aggregating the retweeing *E* ⤏ *E* links between pairs of organizations, opinion leaders, and individuals, as a result of their following relationship, a weighted link in generated for each case. Among these links, the combination of organizations to opinion leaders (only 6% of the total communication flow) and thence to individuals (only 7% of the total communication flow), which corresponds to the two-step flow of communication pattern, revealed a weak link. Thus, we reject the first null hypothesis (H1). In diffusion networks of energy saving practices, the communication pattern is heterogeneous, and the two-step flow of communication theory is not predominantly describing the flow of communication.

Overall, the greatest participation consisted of the self-sharing loops among organizations retweeting the energy saving practices initiated by each other (54%). Similar self-sharing loops of communications were insignificant in comparison for opinion leaders (4%), and individuals (2%). The second highest share, in this case the highest participation by individuals, was observed when retweeting from organizations (25%). We observed only an insignificant percentage of share of information from opinion leaders and individuals to the organizations (1%).

**Fig 5:**
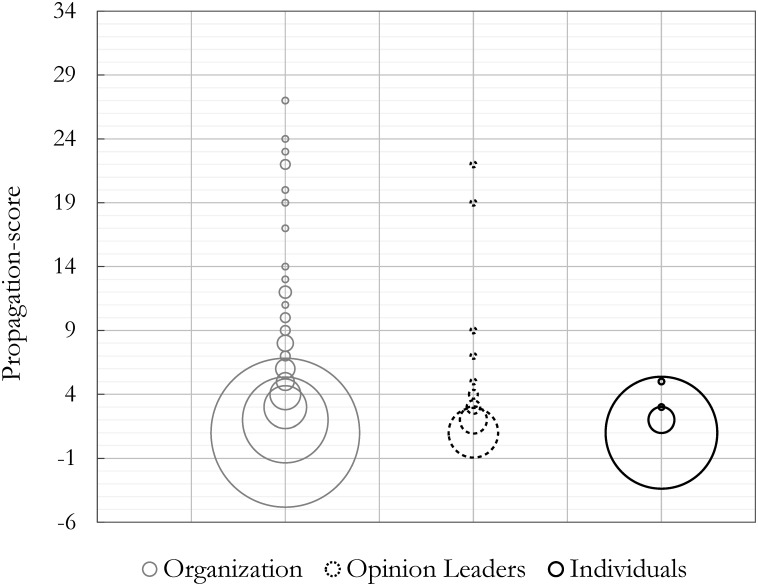
Propagation-scores: Influence breadth. Size of the circles indicate how frequent each score has been observed within an entity group of organization, opinion leader, or individual.

**Fig 6:**
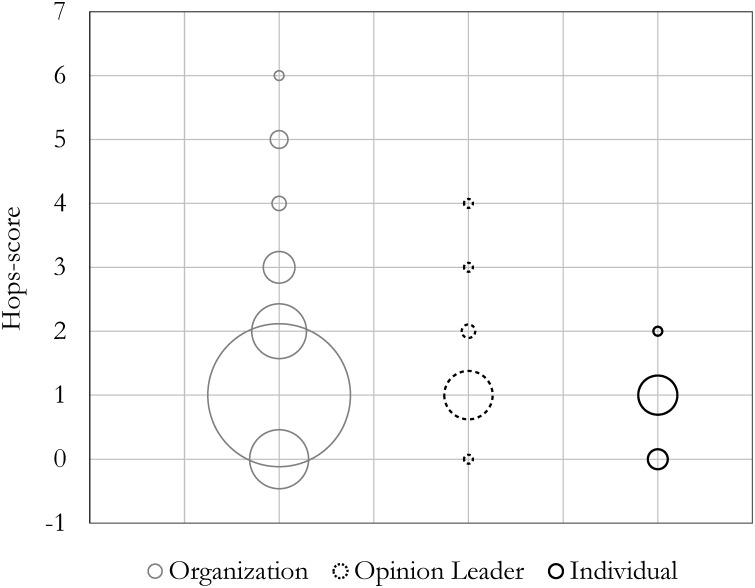
Hop-scores: Influence depth. Size of the circles indicate how frequent each score has been observed within an entity group of organization, opinion leader, or individual.

Further inspection of the empirical distribution of the level of influence for each entity type in the communication network (Figs 5 and 6) revealed that organizations exhibited the highest levels in both cases (with the highest propagation-score, 27, and the highest hop-score, 6). The size of the circles in Fig 5 represent the count of each score and the distribution of the propagation-scores for each entity type within the network is an indicator for their breadth of influence. Fig 6 uses the number of hops traveled by each entity’s tweets and retweets as an indicator for the depth of their influence within the network.

Although organizations may appear to have the highest influential values in both scenarios, the statistical results from a Mann-Whitney U test, which compares the influential values for each pair, reveals that organizations and opinion leaders have insignificant differences in the breadth and depth of their influence levels. However, both have significantly higher influence values than individuals in breadth and depth within the network. This can be explained by examining the data presented in Fig 4, which shows that individuals are mostly influenced by organizations and make the highest proportion of retweets from them. Hence, we find that mass interpersonal communicators (i.e. individuals) are not necessarily most influenced by personal influence from mass personal communicators (i.e. opinion leaders) in the diffusion of energy practices. Finally, we observed that the flow of energy practice diffusion generally originates from the most influential communicators (i.e. organizations or opinion leaders) and then diffuses to the rest of the population. Despite the emergence of a WOM communication network, the anticipated interpersonal propagation of information and influence failed to reveal substantial development between individuals. To investigate the entity heterogeneity property of the network, we therefore analyzed the ‘who-told-whom’ network of energy communication.

The results of the statistical analysis evaluating the breadth (i.e., *p = 6.09×10^−17^*) or depth (i.e., *p = 1.34×10^−16^*) of influence for each entity type using a Kruskal-Wallis test [70] identified a significant difference among the influence values of organizations, opinion leaders, and individuals (Table 1). As noted above, although organizations may appear to have the highest influential values in both scenarios, from the statistical results of a Mann-Whitney U test [71] we were not able to identify any significant difference between the influence values of organizations and opinion leaders for either breadth (*p = 0.478*) and depth (*p = 0.484*), yet significantly higher than individuals within the network. Thus, the null hypothesis for H2a, which assumes opinion leaders are not the most influential entities in the diffusion network, is rejected, suggesting no significant domination in terms of influence by this group. Both organizations (*p < 1×10*^−06^) and opinion leaders (*p = 6.4×10*^−05^, and *p = 7.3×10*^−05^) have greater influence in breadth and depth compared to individuals (Tables 1 and 2). Further, Pearson’s Chi-square test detected significant difference in individuals being influenced by other entities according to the diffusion flow among each pair. We reject the Pearson’s Chi-square null hypothesis that individuals are being equally influenced by organizations, opinion leaders, and other individuals independent of who the influential entity is. Therefore, the null hypothesis for H2b, which suggests that individuals are not most influenced by opinion leaders in the diffusion network, is rejected. Table 3 details the statistic of this test, suggesting that the individuals are in fact most influenced by organizations (73.53%).

**Table 1:** Comparison of influential values in breadth (*propagation-score*), and depth (*hop-score*). Results from Kruskal-Wallis Test (H2).

	Propagation-score			H-score		
	Org.	Op.L.	Ind.	Org.	Op.L.	Ind.
Mean rank	2.05	2.23	1.08	0.48	0.46	0.069
*H*	74.68			73.09		
*p*	6.09*x*10^−^17***			1.34*x*10^−^16***		

*** p < 0.001

**Table 2:** Comparison of influential values in breadth (*propagation-score*), and depth (*hop-score*). Results from Mann-Whitney U Test (H2).

	Propagation-score			H-score		
	Org.-Op.L.	Org.-Ind.	Op.L.-Ind.	Org.-Op.L.	Org.-Ind.	Op.L.-Ind.
*U*	20227.5	20227.5	10305.5	20207.5	86857.0	10264.0
*p*(*one*-*tailed*)	0.478	< 1*x*10^−^06***	6.4*x*10^−^05***	0.484	< 1*x*10^−^06***	7.3*x*10^−^05***

*** *p* < 0.001

**Table 3:** Comparison of diffusion flow from organizations, opinion leaders, and individuals to individuals. Results from Pearson’s Chi-square Test (H2b).

	Influential Entity		
	Org.	Op.L.	Ind.
Expected	33.33%	33.33%	33.33%
Observed	73.53%	20.59%	5.88%
*df*	2		
*χ*^2^	25.826		
*p*	< 0.0001***		

*** *p* < 0.001

## Discussion

### Endogenous vs. Exogenous Diffusion of Energy Saving Practices

The role of large online heterogeneous social networks in influencing public opinion on energy conservation and technology topics is poorly understood and lacks a detailed empirical analysis of the underlying processes involved in the diffusion of this type of information. Therefore, understanding whether the diffusion of energy saving practices in large online networks is an endogenous diffusion process identical to non-commercial WOM and thus emerges naturally within the network; or an exogenous process that is purposely imposed on the network from committed organizations under marketing control, which could be a biased mechanism, is of utmost importance. Our findings suggest that the diffusion of energy saving practices in large online networks is predominantly exogenous. The extremely low density of this energy-related tweets network indicates that the number of interpersonal links is as yet inadequate for the emergence of an endogenous diffusion of energy saving practices. Although emerged from a ‘following’ relationship that represents social ties, a diffusion network of energy information sharing did not correspond to interpersonal interactions between individuals; Communications between individuals were in fact among the least frequently occurring communications. Results from the communication demographics of a Who-told-whom network identified the causes of dispersion and revealed the diffusion network of energy saving practices in large online networks to be predominantly exogenous and driven by organizations rather than individuals. This finding aligns with Kwak et al.’s study, which also identified the prominence of broadcasters in the Twitter network and suggested the network properties are very similar to those of a news media platform [31].

We found that significant portions of the energy tweets and retweets originated from dedicated accounts belonging to energy conscious companies and organizations. Moreover, organizations represented the most substantial portion of all active entities in the network, with by far the largest share of all the energy tweets and retweets that were communicated in the network. This indicates that the diffusion network of energy saving practice communications did not emerge randomly and was instead initiated by multiple sources of dedicated companies and communities targeting energy efficiency gains. We found that less than 10% of the communications were endogenously generated in the network and the remaining 90% were exogenously generated and thus potentially biased. This defies the ideal diffusion scenario, in which communication is formed naturally among individuals based on their prior experience with the energy products or processes and spreads in the same way as an epidemic.

Although in our sample the diffusion network of energy saving practices was found to be more of a WOM Marketing effort in large online networks, this effort still failed to reach its full potential. Despite the energy saving purposes the organizations are seeking to promote in initiating WOM Marketing, more than 50% of the time, they are self-sharing the energy information and are not meeting their objective of reaching out to individuals or opinion leaders. This implies that the effort involved in initiating WOM communication tends to be more self-reliant and does not simply take advantage of the potential of an existing social network structure. Information and influence are circulating within loops of expert communities and companies rather than traveling through the network. A closer look at the flow distribution of energy saving communications in Fig 1 shows that the upward links to more influential entities from the less credible communicators, or loops within the same entity group (except the organizations group), are minimal to zero density. Yet, a reciprocal communication between organizations and individuals can be observed. This suggests that in the diffusion of energy saving practices within large online networks, entities tend to seek advice from more credible and expert sources than their current hierarchy. For example, our data revealed that when an opinion leader retweeted an energy tweet, he/she was always a follower of a higher-level opinion leader or organization with more expertise in the topic. An opinion leader never retweeted from an individual. Likewise, organizations often seek advice from entities more credible than themselves, which in this case may have resulted in self-sharing loops of energy saving practices. This can also explain why the diffusion of energy saving practices is not endogenously generated, and diffused among individuals. This result supports Troldahl’s fundamental explanation of the role of opinion leaders [72].

### Two-step Flow of Communication

Our findings did not support the initial assumption of a two-step flow of communication, where ideas or information flow from organizations to opinion leaders, who then structure this information to influence individuals. Only a small fraction of the communication flow traveled along the organization-opinion leader-individual path typically seen in the two-step flow of communication model and our empirical data did not support either of its two fundamental assumptions—that opinion leaders make greater use of mass media than individuals, and that they influence individuals more than they are influenced by them. Our results contradicted the two-step flow model, suggesting instead that a direct one-step flow from organizations to individuals outperforms the effect of opinion leaders in the current process. This aligns with the criticism that has been leveled against the two-step flow model. The interpersonal effects between individuals are still very weak. Although, the second highest share of influence was from individuals retweeting messages from (i.e. being influenced by) organizations, more than 95% of the time these were not retweeted again by the recipients. This means even though organizations did mostly communicate to individuals rather than opinion leaders, their propagation-scores remained low and their influential values on individuals in depth were minimal.

These findings suggest that the flow of information and influence in WOM diffusion of energy saving practices does not necessarily follow the two-step flow of communication model. This is either due to the lack of opinion leaders employed by organizations and neglecting their potential influence in promoting energy saving practices, or a lack of real influential values for opinion leaders in propagating energy topics to the wider population. This suggests that, in our context, there is scope for a larger appeal to individuals to engage in energy information initiatives and take action to propagate the offered energy information across their own social contacts. Our findings also imply that in the context of energy, individuals may seek the opinions of others and are more influenced by experts in the field. This aligns with the findings of other studies that found messages of certain topics only being circulated within certain communities [73], and reported that when the public lack sufficient knowledge or desire to make self-judgments or decisions on an issue, they instead rely on trusted sources of information (e.g., experts and scientists) to form an opinion about topics such as climate change [6].

### Influence Propagation

Identifying influential entities is critical for understanding the spread of energy efficient practices, as these entities have the capacity to accelerate or immediately decay propagation. Loops of self-sharing information among the same entity types, in particular when organizations or opinion leaders retweet each other, increases the likelihood of the information reaching individuals, especially if there are a high number of individuals among the organization or opinion leader’s contacts. However, the notion of ‘influence exchange’ rather than just ‘information exchange’, cannot be justified when the flow is from organizations to organizations, or opinion leaders to opinion leaders; an opinion leader will not actually be leading an opinion if it originates from another opinion leader and there will be no resulting change of behavior in the population.

In this study, information delivery can occur either directly or indirectly through mass communicators (organizations), mass personal communicators (opinion leaders), and mass interpersonal communicators (individuals). While all three entities are considered influential communicators, they each have limited social reach, and can thus only expect to directly influence a fraction of the total population. It is therefore critical to understand who is most influential for a given topic and prioritize diffusion strategies that target those entities with higher influence values in breadth and depth. When measuring the breadth and depth of influential values among entities for this study, we looked at the propagation-scores and number of hops traveled by their tweets. While organizations play a key role in energy information propagation, we found that they seldom achieved wide diffusion of energy saving practices. Opinion leaders have been found to mediate the link between organizations (mass media) and individuals, but we found no significantly different influential values for opinion leaders and organizations. By measuring proxies of influence in a large online social network, we discovered that when it comes to the diffusion of energy saving practices, mass interpersonal communicators (individuals) are not equally influenced by different entities but instead are most influenced by direct communication links from mass communicators (organizations). The two-step flow model was originally proposed to explain the unanticipated low influence of mass communication media in presidential elections, contending that mass media sources influence individuals through opinion leaders. However, it appears that where environmentally significant topics such as energy conservation are concerned, the influence exerted by organizations and opinion leaders are equally significant. Although our results do not exclude the possibility that the two-step flow hypothesis is instrumental, the potential benefits from this model may be being neglected. People are influenced by exposure to other sources of energy practice diffusion with high credibility and seek out opinions from energy experts, so energy saving practices are less likely to be diffused extensively without considerable involvement from credible and influential entities in various communities. Targeting the most influential hubs will thus maximize diffusion.

Although there is a confined population involved in the diffusion of energy information sharing, existing interventions are unable to reach a mass audience and induce public opinion change directly; traditional mass communication energy interventions for information sharing are simply not as productive in online social networks. When it comes to pressing societal issues such as divergent and often ill-informed public perceptions of climate change and underestimations about energy conservation where vast and rapid public opinion formation is needed, increasing social engagement with online content becomes critical. Given that climate change and energy conservation issues are best communicated online through mass communications, organizations need to focus more on online engagement with the public to effectively influence and drive a cultural shift in this crucial area. Prioritizing the target audience and selecting appropriate strategies accordingly to avoid the immediate decay of diffusion is thus of paramount importance.

Online social networks such as Twitter are powerful tools for influencing social consciousness, leading to rapid public opinion formation on energy and climate change and reaching a broader and more accurately targeted population at a speed never previously possible. However, they do require the right person or entity to spread the message. Our findings suggest that carefully designed strategies are needed to take full advantage of such capabilities; simply relying on conventional wisdom that identifies opinion leaders as being the most influential is no longer sufficient for today’s web-savvy population. Diffusion in different contexts will have different influential entities, as users’ behavior varies for different situations [74]. In this sense, our study departs from earlier studies in diffusion networks.

Despite the immense potential of online social networks, as yet we have developed only the beginnings of a model to explain how this phenomenon functions. We recommend that social network researchers exploit the opportunities afforded by complex networks analysis to examine structural properties of such online heterogeneous communication networks in temporal dimension [75], detect community structures [76] and characterize the dynamic behavior of the governing patterns [42, 43]. The potential role of online social networks in disseminating energy-related information deserves greater attention and will involve enhanced roles for both organizations and opinion leaders. As our findings reveal, public opinion formation regarding energy and climate change is best achieved through direct communications between organization and individuals. Deliberate consideration of the immediate as well as the long term effects of such communications can increase public awareness and result in substantial public support for policies that address climate change and energy issues, leading to significant environmental benefits.

## Supporting Information

S1 Table Descriptive statistics of Propagation-scores by entity type: Organizations (Org.), Opinion Leaders (Op.L.), and Individuals (Ind.). (TEX)

S2 Table Descriptive statistics of Hop-scores by entity type: Organizations (Org.), Opinion Leaders (Op.L.), and Individuals (Ind.). (TEX)

S1 Fig Display of histograms of scores by entity type. (TIF)

S2 Fig Display of box-and-whiskers plots of scores by entity type. (TIF)
