# Supplementary figures and images for: Diffusion Dynamics of Energy Saving Practices in Large Heterogeneous Online Networks
Source: PLoS One. 2016 Oct 13;11(10):e0164476. doi: 10.1371/journal.pone.0164476 (PMC5063345; doi:10.1371/journal.pone.0164476)

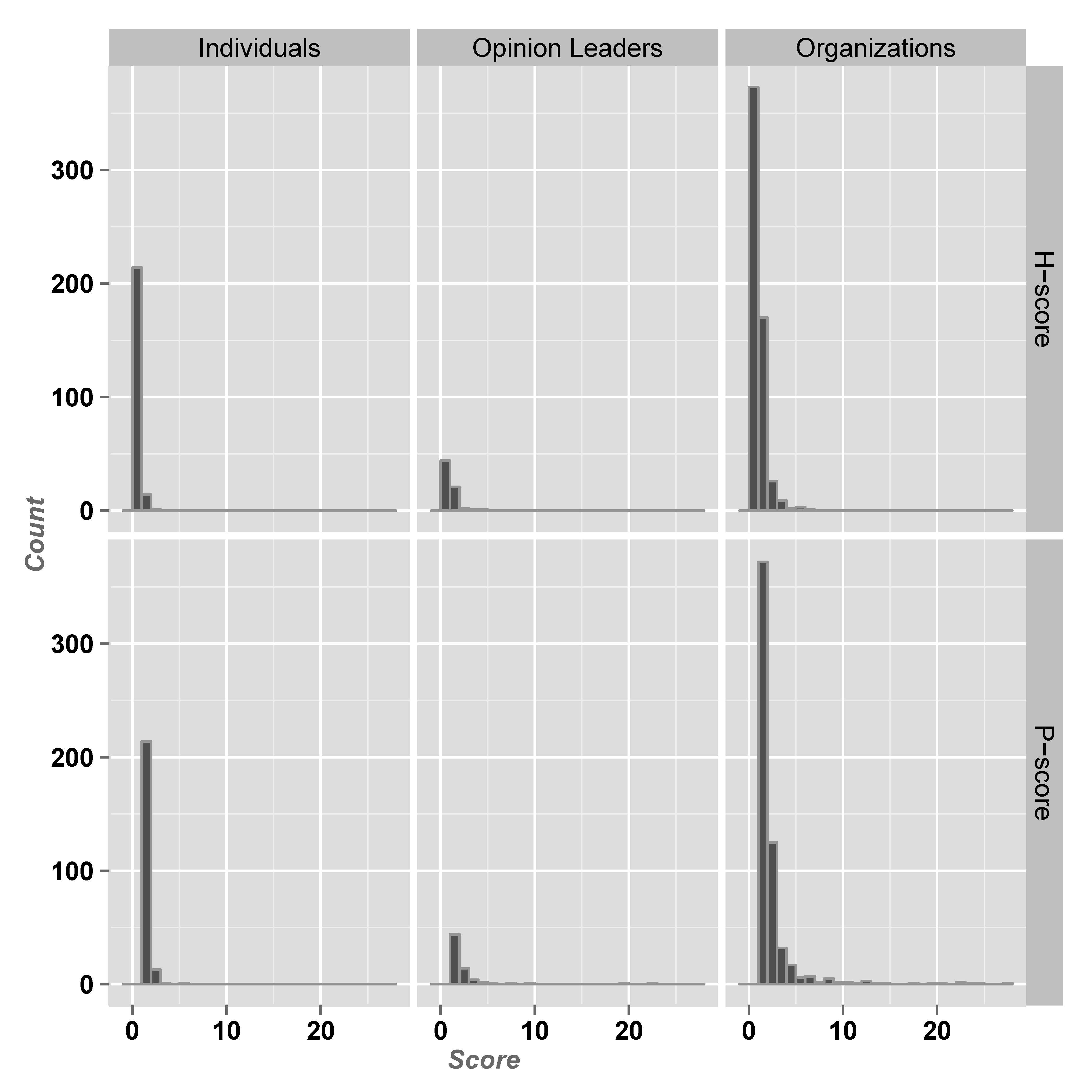

Supplement: S1 Fig — (TIF) [file pone.0164476.s003.tif]

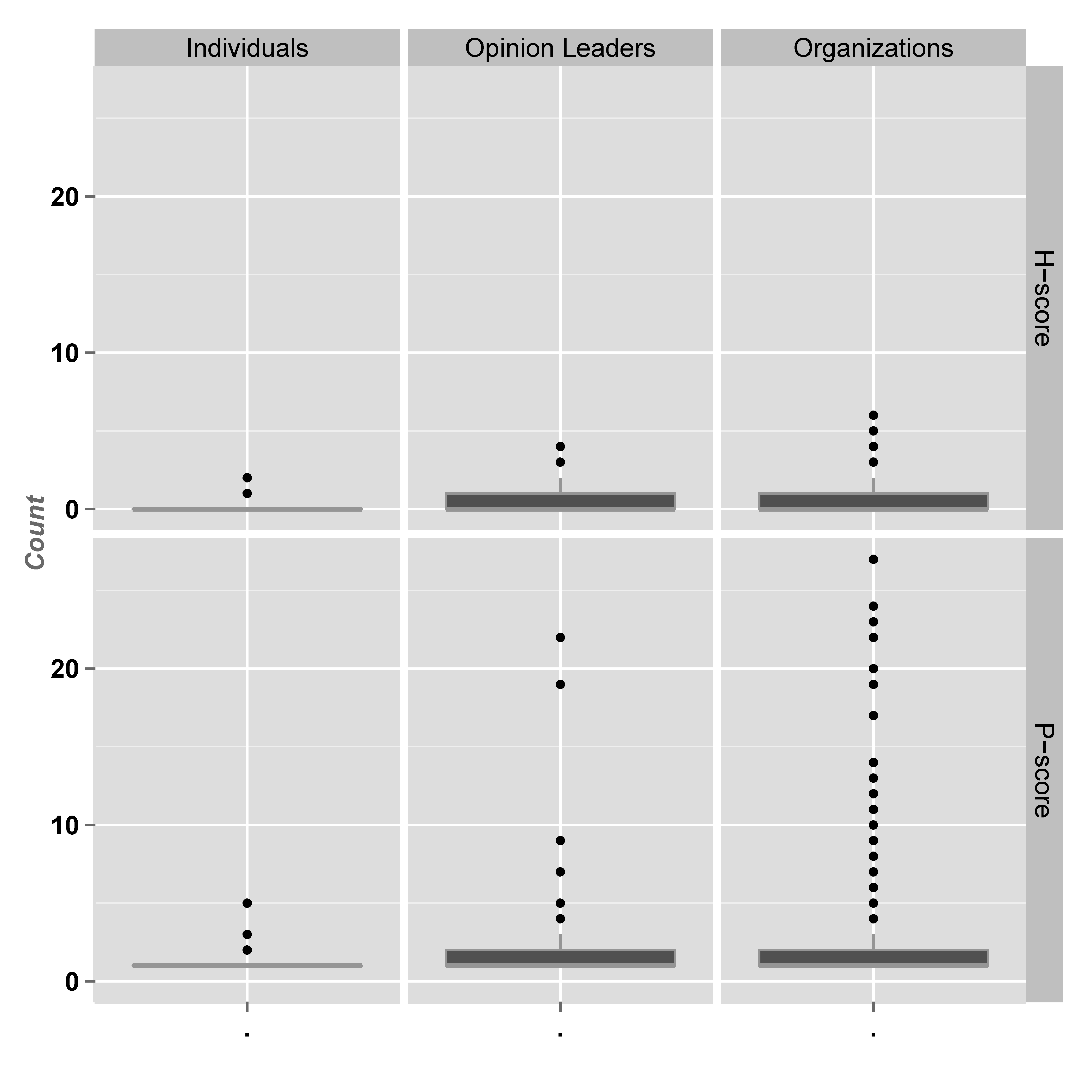

Supplement: S2 Fig — (TIF) [file pone.0164476.s004.tif]
